# A rapid multiplex real-time PCR detection of toxigenic *Clostridioides difficile* directly from fecal samples

**DOI:** 10.1007/s13205-022-03434-6

**Published:** 2023-01-19

**Authors:** Xiao-xi Jia, Yuan-yuan Wang, Wen-zhu Zhang, Wen-ge Li, Lu-lu Bai, Jin-xing Lu, Chao-feng Ma, Yuan Wu

**Affiliations:** 1 State Key Laboratory Infections Disease Prevention and Control, Collaborative Innovation Center for Diagnosis and Treatment of Infectious Diseases, National Institute for Communicable Disease Control and Prevention, Chinese Center for Disease Control and Prevention, Beijing, 102206 China; 2 State Key Laboratory for Infectious Disease Prevention and Control, National Institute for Viral Disease Control and Prevention, Chinese Center for Disease Control and Prevention, Beijing, China; 3 Shaanxi University of Chinese Medicine, Xi’an, 710000 Shaanxi China; 4 Xi’an Center for Disease Control and Prevention, Xi’an, 710000 Shaanxi China

**Keywords:** Multiplex real-time PCR, Toxigenic *Clostridioides difficile*, Toxin a/B, Binary toxin

## Abstract

This study developed a new single-tube multiplex real-time PCR method for detecting toxigenic *C. difficile* directly from fecal samples using *tcdA*, *tcdB*, *cdtB,* and internal gene *tpi* as targets, which could be performed on kinds of polymerase chain reaction device including point-of-care testing (POCT), with improved detection efficiency. The specificity, sensitivity, and repeatability of each gene was evaluated using 69 *C*. *difficile* isolates and 74 fecal samples. Results were compared with established PCR, qPCR, and ELISA methods. Interspecies specificity was 100% based on six common intestinal pathogens (*Escherichia coli, Enterococcus Faecium, Enterococcus faecalis, Clostridium perfringens, Bacteroides fragilis, Clostridium botulinum*). The lower detection limit (LDL) for *tcdA*, *tcdB*, and *cdtB* with pure *C*. *difficile* DNA was 10^1^,10^0^, and 10^0^ copies/μL, respectively, the coefficients of variation among different experimental batches and within each experimental batch were both less than 3%, which shows that this method has strong repeatability. And the LDL of fecal DNA was 5 × 10^0^, 5 × 10^3^, and 5 × 10^2^ colony-forming units (CFU)/g, respectively. In addition, the efficiency for detection of *tcdA* was compared with established PCR and real-time PCR methods, demonstrating high consistency (98.4%) and similar sensitivity. ELISA was used to confirm inconsistent results, which were identical with our method. The sensitivity and specificity for detecting toxigenic *C*. *difficile* in fecal samples were 96.49% and 94.12% compared with the toxigenic culture (TC). This method effectively identified the toxigenic and non-toxigenic strains with high specificity, sensitivity, and repeatability, and could reduce the false positive rate of *tcdA*, and accurately identify the typical Asian strain RT017, making it potentially contribute to the surveillance of CDI in China.

**Supplementary Information:**

The online version contains supplementary material available at 10.1007/s13205-022-03434-6.

## Background

*Clostridioides difficile* is an anaerobic Gram-positive bacillus that is widespread in the intestines of humans and other animals (Johnson et al. 2021; Czepiel et al. 2019). Risk factors such as antibiotic exposure, older age, and weakened immune system, are known to be closely related to *C. difficile* infection (CDI) (Leffler and Lamont 2015), which is one of the most common hospital-acquired infections with symptoms of diarrhea, pseudomembranous colitis, toxic megacolon, and even death (McDonald et al. 2018). A multistate point-prevalence survey of healthcare-associated infections in the United States found *C. difficile* was the most commonly reported pathogen (causing 12.1% of healthcare-associated infections) (Magill et al. 2014), and previous publications which demonstrated the high economic burden of CDI for healthcare settings and health insurance systems (Mollard et al. 2019). In summary, CDI poses a significant burden on both life health and social resources, which should be of great concern.

Toxins produced by *C. difficile* play an important role in the pathogenic process and two major toxins are produced: toxin A (enterotoxin) and toxin B (cytotoxin), which can be produced alone or simultaneously. Moreover, a third toxin-binary toxin is found in some *C*. *difficile* isolates, such as hypervirulent RT027 / NAP1 / BI (Nagy 2018) and RT078. Although the exact role of binary toxin in pathogenesis is unclear, but it is thought to be associated with high incidence, recurrence, and mortality of CDI (Bacci et al. 2011; Stewart et al. 2013). However, typical Asian strain RT017( toxin A-negative toxin B-positive) causing several outbreaks worldwide, has also been identified in recent years (Drudy et al. 2007).

There are three commonly used methods for the identification of toxigenic *C. difficile*: (i) TC (toxigenic culture) is a two-step method that combined with *C. difficile* culture and cell cytotoxicity assay (CCNA) (Nagy 2018); (ii) enzyme immunoassay (EIA) for toxin A/B and glutamate dehydrogenase(GDH) (Bagdasarian et al. 2015); (iii) nucleic acid amplification tests (NAATs) targeting toxin-encoding genes, which include PCR, quantitative PCR (QPCR), loop-mediated isothermal amplification (LAMP), and helicase-dependent isothermal DNA amplification (HAD). TC operation is complex and time-consuming, which is mainly used for epidemiological research and evaluation of new methods (Bagdasarian et al. 2015). The sensitivity and specificity of immunological testing can vary, and it must be combined with a high sensitivity-specific approach to make up for its shortcomings. Therefore, NAAT was recommended for diagnosing CDI worldwide in both two-steps and three-steps diagnosis procedures (Lee et al. 2021).

In the present study, our novel method can simultaneously detect *tcdA*, *tcdB*, and *cdtB* genes, which improves the accurate identification of toxigenic *C. difficile* isolates directly from fecal samples. First, it is noteworthy that the typical Asian strain RT017, which has an *A-B* + *cdtA/B-* profile, plays an important role in CDI epidemiology in China. A deletion of 1821 bp was found in the repeat region of *tcdA*. Although none toxin A was produced due to the presence of a stop codon in these *A-B* + isolates, but there still are DNA fragments remaining in *tcdA-*negative isolates (Rupnik et al. 2003), primers that circumvent this deleted region may lead to false-positive results for toxinA in this type strain. Second, other multiple qPCR methods only included *tcdA* and *tcdB* as target genes, but binary toxin genes were not included (Luna et al. 2011; Kubota et al. 2014; Bélanger et al. 2003). Additionally, since binary toxin genes *cdtA* and *cdtB* may be fused, choosing *cdtA* as the target gene may lead to false-negative results for binary toxin (Gerding et al. 2014). In other previously published multiple qPCR methods, *cdtA* rather than *cdtB* was selected as the target gene for binary toxin detection, which may lead to inaccurate detection of binary toxin (Kilic et al. 2015; Hoegh et al. 2012). Therefore, *cdtB* was selected as the target gene for binary toxin detection.

In a word, a multiplex real-time PCR method was developed to detect toxigenic *C. difficile* directly from fecal samples, involving *tcdA*, *tcdB*, *cdtB,* and internal gene *tpi* as targets, which could be performed on kinds of polymerase chain reaction devices including POCT for rapid detection and identification of clinical CDI cases. Furthermore, this method could reduce the false positive rate of *tcdA*, accurately identify the typical Asian strain RT017 with improved detection efficiency, making it potentially contribute to the surveillance of CDI in China.

## Methods

### Design of primers and probes

Complete sequences of *tcdA* (NZ_FUUL01000004.1, NC_017178.1, NZ_CDND01000001.1, NC_017174.1, NC_013316.1), *tcdB* (NZ_FUUL01000004.1, NC_017178.1, NZ_CDND01000001.1, NC_017174.1, NC_013316.1), and *cdtB* (AF271719.1, HQ639679.1, FN538970.1, NC_017178.1, NZ_CDND01000001.1, NC_017174.1)were download from NCBI GenBank entries and aligned to determine the conserved regions. According to the consensus sequences, primers and probes were designed by Primer Express software v.3.0 (Applied Biosystems, Foster City, CA), and then were BLAST in GenBank to test the specificity. There was no crossover with other pathogens using BLAST searches in the NCBI database. All primers and probes were included in patent application CN202010825309. X. RT017 is a typical Asian strain characterized by a partial deletion in the repeat region of the *tcdA* gene. Due to the presence of a termination codon, it does not produce toxin A, but most of the *tcdA* negative isolates still possess remaining DNA fragments. Therefore, it is necessary to design primers targeting the absent region of the *tcdA* gene for the accurate identification of toxin A.

### Reaction system and parameters

The optimized qPCR reaction system (20 μl) is composed of 10 μl Premix (TaKaRa RR390A), 0.4 μl of 10 mM forward and reverse primers (single-tube multiplex), 0.8 μl of 10 mM probe, 0.2 μl of Rox Reference Dye II, 2 μl of template DNA, and 6.2 μl of deionized water. The two-step method was employed by heating at 95 °C for 20 s, followed by 40 cycles at 95 °C for 3 s and 58 °C for 30 s. Reactions were performed on a 7500 Fast Real-Time PCR System (Applied Biosystems), LightCycler 96 (Roche), QuentGene 9600(Bioer), and iPonatic(Sansure).

### Construction of standard plasmids and standard curves for the three genes

The *C*. *difficile* isolate ATCC-BBA1803 (RT027) was used to amplify the three target toxin genes. PCR products were purified using an EasyPure Quick Gel Extraction Kit (Trans, China), and then ligated into the PMD18-T vector (TaKaRa, Japan), which were subsequently transformed into JM109 competent cells (TaKaRa). Positive plasmids were identified by Blue-White Screening of positive colonies and sequencing of PCR products from plasmids. The standard plasmid concentration was obtained according to the copy number conversion formula (6.02 × 10^23^copies/M × (concentration)/(MWg/mol) = copies/ml), and ten-fold dilutions of the standard into ten gradients were used as templates to prepare the standard curve.

### Evaluation of the specificity, sensitivity, and repeatability of qPCR for pure bacterial DNA

Standard strains of another six common intestinal pathogens (*Escherichia coli, Enterococcus Faecium, Enterococcus faecalis, Clostridium perfringens, Bacteroides fragilis, Clostridium botulinum*) were used to test interspecies specificity (Table S1). A total of 69 *C*. *difficile* isolates with different toxigenic types, including eight ATCC standard strains and 61 clinical isolates, were used to verify intraspecies specificity (Table S1). Through standard curve analysis, the pure bacterial lower detection limit (LDL), also known as the minimum concentration of detected plasmid, was determined. To assess the repeatability of this method, different concentrations (high, medium, and low copies) of three standard plasmids harboring *tcdA*, *tcdB*, and *cdtB* genes were tested in triplicate every other day. Then three parallel DNA samples were tested to obtain the coefficient of variation between batches and the coefficient of variation within groups (Table S2).

### Preparation and detection of C. difficile-simulated fecal samples

A pure clone of the *C. difficile* ATCC1803 toxigenic strain was picked and mixed with 1 ml BHI (Brain Heart Infusion Broth) to achieve an McF 0.5 suspension of ~ 10^5^ colony-forming units (CFU)/ml by the standard plate counting method. Bacterial suspensions at 10^0^ to 10^5^ CFU/ml were obtained by serial dilution, and 0.1 ml of each suspension was transferred into 0.2 mg *C. difficile*-negative fecal samples from healthy individuals at a final concentration of 5.0 × 10^− 1^ to 5.0 × 10^4^ CFU/g. Total DNA was extracted from these simulated fecal samples for qPCR evaluation of LDLs for the three target genes (*tcdA*, *tcdB*, and *cdtB*; Table 1).

**Table 1:** Lower limit of detection of three target genes in simulated feces

Target genes	CFU/g	5.0 × 10^4^	5.0 × 10^3^	5.0 × 10^2^	5.0 × 10^1^	5.0 × 10^0^	5.0 × 10^− 1^
tcdA	Sample	26.48415	29.63562	33.04662	36.79008	36.71255	Undetermined
	Replication1	26.55865	29.64664	32.28347	37.06828	36.01574	Undetermined
	Replication2	26.47834	29.70147	32.97718	36.2962	36.13764	36.60099
	SD	0.044782	0.035268	0.42199	0.391029	0.37214	
tcdB	Sample	31.4739	34.99224	Undetermined	Undetermined	Undetermined	Undetermined
	Replication1	31.56133	35.34561	38.45827	Undetermined	Undetermined	Undetermined
	Replication2	31.53615	34.95603	37.68927	Undetermined	Undetermined	Undetermined
	SD	0.045007	0.215232				
cdtB	Sample	28.53067	31.93304	35.61412	39.17686	Undetermined	Undetermined
	Replication1	28.38683	31.92636	35.62996	Undetermined	Undetermined	Undetermined
	Replication2	28.45992	31.87342	34.94851	38.94106	Undetermined	Undetermined
	SD	0.071922	0.032662	0.388941			

### Detection of human fecal samples using multiplex real-time PCR

A total of 74 frozen fecal samples in our laboratory were used to evaluate our qPCR detection method. A Fecal DNA Extraction Kit (TIANGEN) was used to extract fecal DNA according to the manufacturer’s instructions. The results were compared with those of the gold standard (TC) method using *tpi* gene as internal control to evaluate the performance (Table 2).

**Table 2:** Fecal samples included in this study and test results

Number	Samples	Toxigenic	Detection result			
			tcdA	tcdB	cdtB	tpi
1	10,005	N	−	−	−	+
2	11,032	N	−	−	−	+
3	24,078	Y	+	+	−	+
4	25,049	Y	+	+	−	+
5	0204–001	N	−	−	−	+
6	0204–005	Y	+	+	−	+
7	0205–005	N	−	−	−	+
8	0205–007	N	−	−	−	+
9	0205–009	N	−	−	−	+
10	01,047	Y	+	+	−	+
11	12,038	Y	+	+	+	+
12	25,047	Y	+	+	−	+
13	25,049	Y	+	+	−	+
14	20,051	Y	+	+	−	+
15	25,053	Y	+	+	+	+
16	25,058	Y	+	+	+	+
17	0201–016	Y	+	+	−	+
18	0201–021	Y	+	−	−	+
19	0201–033	Y	+	+	−	+
20	0201–045	Y	+	+	−	+
21	0201–059	Y	+	+	−	+
22	0201–069	Y	+	+	−	+
23	0201–074	Y	+	+	−	+
24	0201–077	Y	+	+	−	+
25	0204–004	Y	−	−	−	+
26	0205–002	Y	−	+	−	+
27	0205–006	Y	+	+	−	+
28	0206–005	Y	−	+	−	+
29	0207–002	Y	+	+	−	+
30	10,122	N	−	−	−	+
31	10,010	Y	+	−	−	+
32	11,034	Y	+	+	−	+
33	20,086	N	+	−	−	+
34	24,078	Y	+	+	−	+
35	0201–018	Y	−	+	−	+
36	0201–029	Y	+	+	−	+
37	0201–041	Y	+	+	−	+
38	0201–080	Y	+	−	−	+
39	0203–004	Y	+	+	−	+
40	0203–006	Y	−	+	−	+
41	0205–001	Y	+	−	−	+
42	0205–008	Y	−	+	−	+
43	0206–003	Y	+	+	−	+
44	0207–003	Y	−	−	−	+
45	0207–006	Y	+	+	−	+
46	0208–002	N	−	−	−	+
47	0208–003	Y	+	−	−	+
48	10,007	N	−	−	−	+
49	10,122–2	N	−	−	−	+
50	21,076	N	−	−	−	+
51	22,012	N	−	−	−	+
52	29,033	N	−	−	−	+
53	0204–001	N	−	−	−	+
54	21,074	Y	+	+	−	+
55	12,038–2	Y	+	+	+	+
56	01,047–2	Y	+	+	−	+
57	11,068	Y	+	+	−	+
58	10,115	Y	+	+	−	+
59	20,051–2	Y	+	+	−	+
60	20,054	Y	+	+	−	+
61	21,078	Y	+	+	−	+
62	25,047	Y	+	+	+	+
63	25,058–2	Y	+	+	+	+
64	09,066	Y	+	+	+	+
65	12,038–2	Y	+	+	+	+
66	25,053–2	Y	+	+	+	+
67	0201–006	Y	+	+	−	+
68	0201–014	N	−	−	−	+
69	0201–038	Y	−	+	−	+
70	0201–040	Y	+	+	−	+
71	0201–063	N	−	−	−	+
72	0201–071	Y	+	+	−	+
73	0207–009	Y	+	+	−	+
74	0208–001	Y	+	+	−	+

Letter Y refers to positive results and N means negative results. The red letters refer to inconsistent results

### Comparison with previously reported detection methods for tcdA and confirmation by ELISA for TcdA

Two pairs of classic PCR primers for gene *tcdA* (Kato et al. 1999; Lemee et al. 2004) were compared with our method using 33 *C*. *difficile* RT017 isolates and 30 other types of clinical isolates preserved in our laboratory (Table S3). For inconsistent results, ELISA was employed for clarification. Briefly, negative control (ATCC43593), positive control (ATCC-BBA1803), and tested *C*. *difficile* (N20) isolates (Table S3) were anaerobically cultured in BHI liquid medium at 37 °C for 24 h. After centrifugation, the supernatant was taken and filtered through a 0.45 μm filter, then incubated overnight at 4 °C in a 96-well plate. The plate was washed with PBST(Phosphate Buffered Saline + Tween) and blocked with 5% bovine serum albumin at 37 °C for 2 h. Polyclonal antibody (List Biological Laboratories, Toxin A IgY, 1:1000 dilution) was used for the first hybridization, and horseradish peroxidase (HRP-conjugated goat anti-chicken IgY (Invitrogen, 1:5000 dilution) served as the secondary antibody. Finally, the results were determined using an EL-TMB Chromogenic Reagent Kit (SANGON, China) at an absorbance of 450 nm. Standard curves were prepared using Toxin A (List Biological Laboratories) as a positive control at an initial concentration of 1600 ng/ml that was diluted twice to 0.05 ng/ml. The absorbance ratio (P/N) of the tested sample (N20) and negative control (ATCC43593) was calculated, and results were considered negative at P/N < 1.5, suspicious at P/N ≥ 1.5 and < 2.1, and positive at P/N ≥ 2.1.

**Fig. 1:**
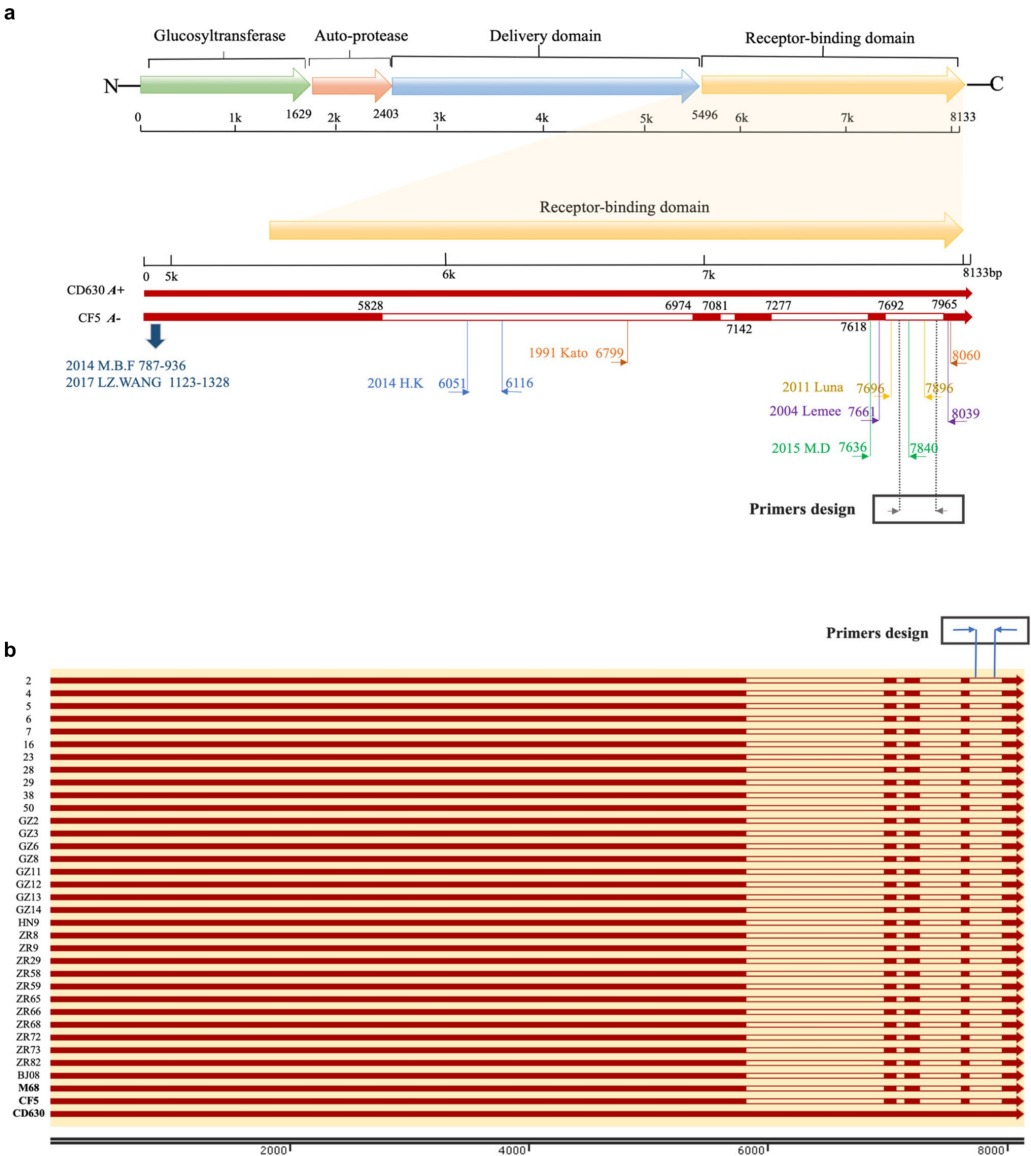
Schematic structure of the *tcdA* gene and the locations of primers in both the schematic diagram and *tcdA* sequences of 33 previously reported RT017 isolates. **a** Schematic structure of the *tcdA* gene and the locations of primers synthesized and tested in this study. **b** Complete sequences of *tcdA* extracted from whole genomes of 33 RT017 isolates and the locations of primers were synthesized and tested in this study. M68 (NC_017175.1), CF5 (NC_017173.1) and CD630 (NC_009089.1) were used as reference strains

In addition, our results were also compared with two previously reported qPCR methods (Luna et al. 2011; Kubota et al. 2014), in which our primers for gene *tcdA* were located in the missing region to avoid potential false positives (Fig. 1a). The qPCR conditions and mixture were previously described. The lower limits of these three pairs of primers for *tcdA* were tested and compared by testing the *C*. *difficile*-simulated fecal samples (Table 3) prepared as described above.

**Table 3:** The results and comparison of three pairs primers for *tcdA* detecting the *C*. *difficile*-simulated fecal samples

Target (annealing temperature)	CFU/g	5.0 × 10^4^	5.0 × 10^3^	5.0 × 10^2^	5.0 × 10^1^	5.0 × 10^0^	5.0 × 10^− 1^
Our tcdA (58℃)	Sample	26.48415	29.63562	33.04662	36.79008	36.71255	Undetermined
	Replication1	26.55865	29.64664	32.28347	37.06828	36.01574	Undetermined
	Replication2	26.47834	29.70147	32.97718	36.2962	36.13764	36.60099
	SD	0.044782	0.035268	0.42199	0.391029	0.37214	
Luna 2011 (57℃)	Sample	26.76287	29.99077	33.65609	37.25234	39.6127	38.66362
	Replication1	26.67333	30.12401	33.5074	36.2081	Undetermined	Undetermined
	Replication2	26.61492	30.10385	33.64701	37.33664	38.74393	Undetermined
	SD	0.07452	0.071819	0.083344	0.628642		
HK 2014 (56℃)	Sample	26.14689	29.84816	33.71031	37.32001	38.19745	39.58826
	Replication1	26.43128	29.70106	33.79443	37.72804	39.00477	39.58448
	Replication2	26.21063	29.72093	33.90454	37.76967	38.14799	39.7678
	SD	0.149239	0.079814	0.097404	0.248465	0.48102	0.104765

### Flow chart

**Fig. 2:**
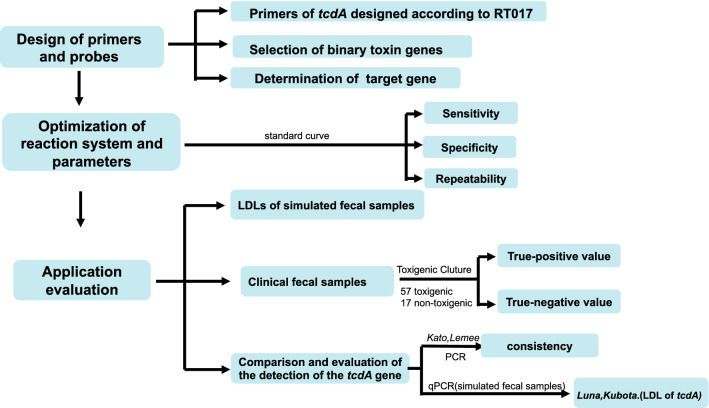
A flow chart of the manuscript

A flow chart for better understanding of the manuscript (Fig. 2).

## Results

### Primers and probes designed in this study

The sequences of the *tcdA* gene in the NCBI database for isolates CD630 (A +) and CF5 (A-) were compared, and four deleted and repeated regions of *tcdA* spanning 1821 bp were identified (Fig. 1a): 5828–6974 (1146 bp), 7081–7142 (61 bp), 7277–618 (341 bp), and 7692–7965 (273 bp). Our primers and probes are located in the deleted region spanning 7692–7965 bp (Fig. 1a). Meanwhile, by blasting against the whole genome sequences of 33 clinical *C*. *difficile* isolates (RT017) reported in our previous work (Wu et al. 2019), primers and probes for *tcdA* in the present study are all within the deleted regions of these 33 strains (Fig. 1b), which further confirmed their accuracy theoretically.

### The real-time PCR method is specific, sensitive, and repeatable

**Fig. 3:**
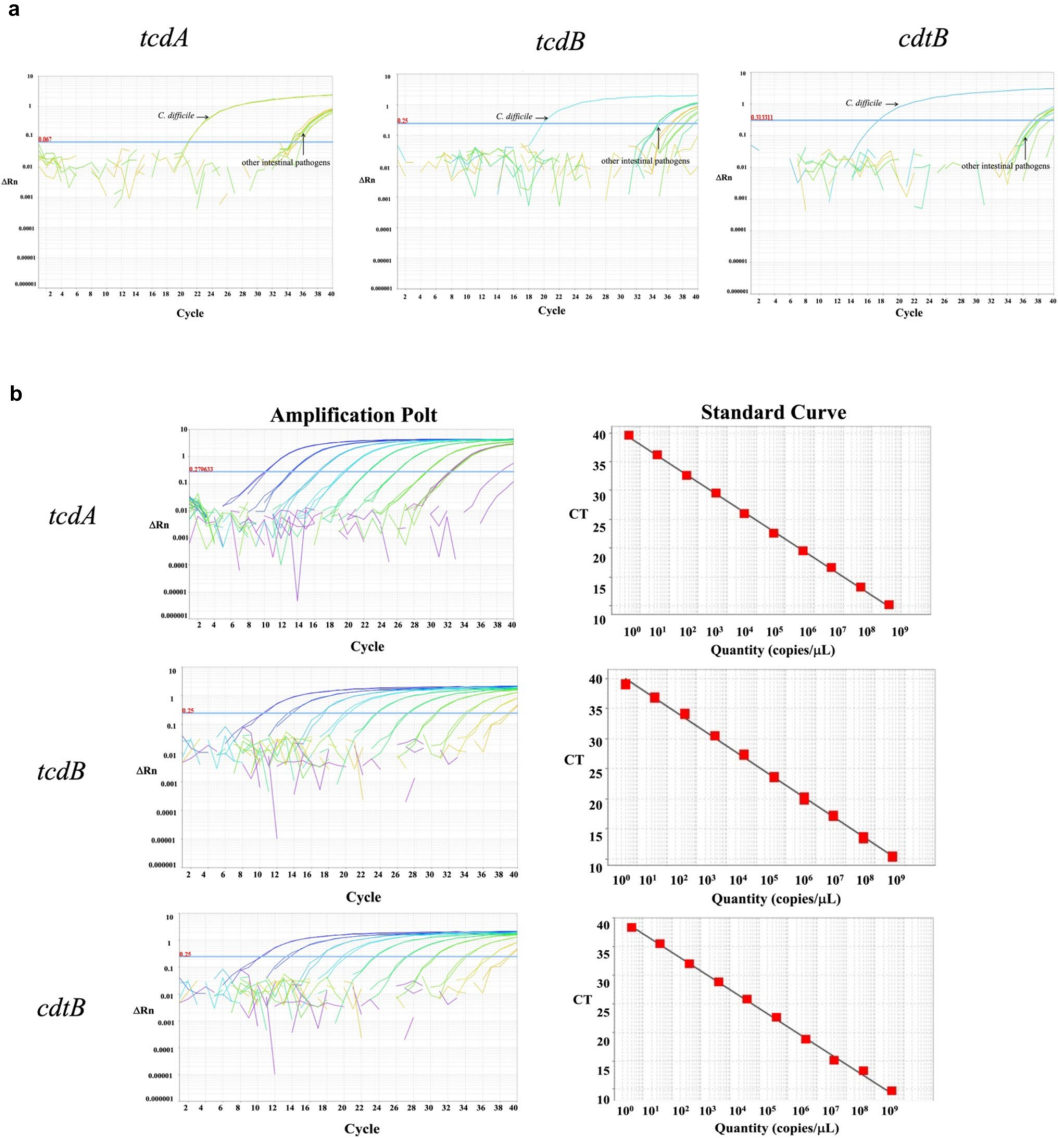
Specificity and sensitivity of the developed qPCR method for detecting *tcdA*, *tcdB*, and *cdtB*. **a** The specificity for detecting the three target genes was determined by comparing them with another six intestinal bacteria. **b** Standard curves and amplification curves for the three target genes

Standard strains of the other six common intestinal pathogens were tested using our primers and probes, and no specific products were amplified (Table S1). ATCC Standard strains of different toxigenic types of *C. difficile* were used to assess intraspecies specificity, and the results were consistent with the defined toxin profiles (Table S1). According to the above results, the specificity of this method was 100%, confirming its ability to detect toxigenic *C. difficile* strains accurately and selectively (Fig. 3a).

Standard curves of the three target genes (*tcdA*, *tcdB*, and *cdtB*) were constructed using plasmids diluted tenfold as templates for qPCR detection (Fig. 3b). The three plasmids were linearly correlated with their corresponding CT (Cycle threshold) values at 10^0^ − 10^9^ copies/μl. Using the lowest concentration of each plasmid that could be detected as the LDL, we verified that the detection limits of DNA from *C. difficile* isolates for *tcdA*, *tcdB*, and *cdtB* were 10^1^, 10^0^, and 10^0^ copies/μl, respectively. PCR amplification efficiency for each gene was 102%, 103%, and 102%, respectively, and correlation coefficients reached 0.998 (Fig. 3b).

To evaluate the repeatability of this method, coefficients of variation among different experimental batches were calculated. Three replicate samples for each gene within each experiment batch were also tested. The coefficients of variation among different experimental batches and within each experimental batch were both less than 3%, which shows that this method has strong repeatability (Table S2).

### Detection of toxigenic C. difficile in simulated fecal samples

When 0.2 g of feces was mixed with 10^0^ CFU bacteria, the method could stably detect *tcdA*, and the bacteria concentration in feces was 5.0 × 10^0^ CFU/g. Gene *tcdB* could be stably detected when 0.2 g of feces was mixed with 10^3^ CFU bacteria, and the bacterium content in feces was 5.0 × 10^3^ CFU/g.

And when 0.2 g of feces were mixed with 10^2^ CFU bacteria, the method could stably detect *cdtB*, and the bacteria content in feces was 5.0 × 10^2^ CFU/g (Table 1). Therefore, the LDL of simulated fecal samples for the three genes was 5 × 10^0^, 5 × 10^3^, and 5 × 10^2^ colony-forming units (CFU)/g, respectively.

### The real-time PCR method detects toxigenic C. difficile in human fecal samples

A total of 74 fecal samples, including 57 samples with toxigenic *C*. *difficile* and 17 samples with non-toxigenic *C*. *difficile*, all confirmed by TC, were randomly selected to evaluate our method (Table 2). Using our method, we detected 56 toxin-positive samples, including one false-positive, and 18 toxin-negative samples, including two false-negative (Table 2). Therefore, the true-positive and true-negative values were 96.49% (55/57) and 94.12% (16/17), respectively.

### Evaluation of the detection of the tcdA gene

**Fig. 4:**
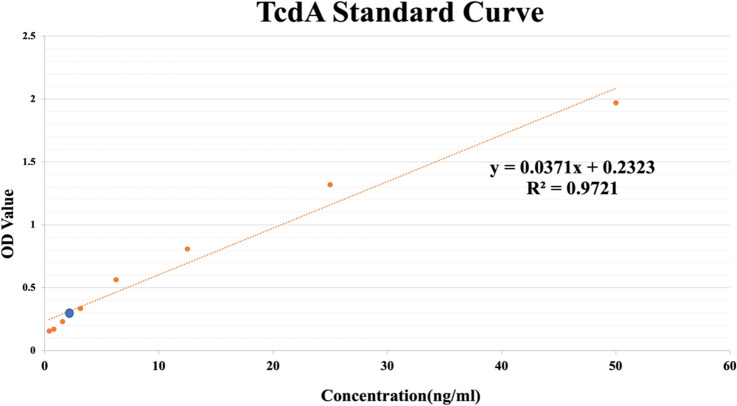
The toxin A in N20 is determined by ELISA. The concentration of toxin A in isolate N20 is indicated by a blue circle

The developed method demonstrated high consistency (98.4%) with two sets of standard PCR primers reported previously for detecting *tcdA* in 63 *C*. *difficile* isolates, and only one strain (N20) yielded different results (Table S3). Therefore, the gold standard ELISA method for detecting toxin A in isolate N20 was used for further confirmation, from which the standard curve shows that the concentration of TcdA was linear at eight concentrations (0.4 ng/ml, 0.8 ng/ml, 1.6 ng/ml, 3.2 ng/ml, 6.4 ng/ml, 12.8 ng/ml, 25 ng/ml, and 50 ng/ml). The ratio of the absorbance between N20 and the negative control, P(0.363)/N (0.15), was 2.42, confirming that isolate N20 is a TcdA-positive strain, with a corresponding toxin A concentration was 3.53 ng/ml (Fig. 4). The ELISA results support this study’s detection results for the tcdA gene.

## Discussion

Toxigenic culture and CCNA (cell cytotoxicity assay) are considered the gold standards for CDI detection (Crobach et al. 2009). However, the CCNA procedure is complicated, time-consuming, and limited by cell types and equipment (Buss et al. 2015). Nowadays, immunological techniques are used as the preliminary screening method for CDI, but the sensitivity and specificity can be unstable and the results must be interpreted together with those of PCR approaches (Guery et al. 2019). In recent years, NAAT has been recommended for auxiliary diagnosis of CDI cases due to its high sensitivity, specificity, and time-saving. To date, at least 15 kinds of *C. difficile* nucleic acid detection methods have been approved by the United States Food and Drug Administration (FDA), of which 12 methods rely on specific instruments, 6 methods only detect the *tcdB* gene, and no methods detect *tcdA*, *tcdB*, and *cdtB* simultaneously (https://www.fda.gov/medical-devices/vitro-diagnostics/nucleic-acid-based-tests).

Our novel method can simultaneously detect *tcdA*, *tcdB*, and *cdtB* genes, which improves the accurate identification of toxigenic *C. difficile* isolates directly from fecal samples. In four previously reported qPCR methods, primers for *tcdA* were not located in the deletion region (Fig. 1a) (Kilic et al. 2015; Hoegh et al. 2012; Avbersek et al. 2011), which might cause the inaccurate identification of *tcdA-*negative isolates including RT017/ST37. In the present study, primers for *tcdA* were situated in the deletion region (7692–7965; Fig. 1a), and results with these primers were highly consistent (98.4%) with those of standard PCR primers reported previously for detecting *tcdA* in 63 *C*. *difficile* isolates, except for only one strain (N20) showing different results (Lemee et al. 2004; Kato et al. 1999) (Table S3). ELISA of the inconsistent N20 strain confirmed the results for our method (Fig. 4).

According to previously reported qPCR methods for detecting toxigenic *C*. *difficil*e isolates, two pairs of *tcdA* primers from *Luna* and *Kubota* are located in the missing region, which were selected and compared with our method. The LDLs for these three qPCR methods were compared using simulated fecal samples. The LDL for *tcdA* in fecal samples was 2.5 × 10^2^ CFU/ml according to *Luna *et al., while the LDL reported by *Kubota *et al*.* was 10^3^ cells/g (Luna et al. 2011; Kubota et al. 2014). The LDLs for the methods of *Kubota* and *Luna* were 5.0 × 10^0^ CFU/g and 5.0 × 10^1^ CFU/g, respectively (Table 3), and our method yielded an LDL comparable with that of the *Kubota* team (Kubota et al. 2014).

In conclusion, we established and validated a real-time PCR method for the detection of toxigenic *C. difficile* simultaneously targeting the *tcdA, tcdB,* and *cdtB* genes. The method displayed good performance for the specificity, sensitivity, and repeatability which has been submitted for a Chinese invention patent (CN202010825309.X). Despite the great advantages mentioned above there are some limitations: the reaction parameters and system should be further optimized to improve the overall sensitivity of the method and to optimize the amplification efficiency, in addition, the sample size should also be increased to obtain more accurate evaluation data.

It is worth mentioning that the method improved the identification of the A-B + straind, including typical Asian strain RT017. It could also be combined with the method in Chinese invention patent CN202010821402.3 for early warning testing of the highly toxigenic strain BI/NAPI/027, and be applicable to CDI epidemiological surveillance and potential outbreak control. In addition, our method is applicable to a variety of fluorescence quantitative PCR instruments, and can also be combined with POCT for rapid detection and identification of clinical CDI cases.

## Conflict of interest

The authors declare that they have no competing interests.

## Supplementary Information

Below is the link to the electronic supplementary material.

Supplementary file1 (PDF 70 kb)

Supplementary file2 (PDF 57 kb)

Supplementary file3 (PDF 63 kb)
