# Supplementary material for: A rapid multiplex real-time PCR detection of toxigenic Clostridioides difficile directly from fecal samples
Source: 3 Biotech. 2023 Jan 19;13(2):54. doi: 10.1007/s13205-022-03434-6 (PMC9849642; doi:10.1007/s13205-022-03434-6)
Supplement: Supplementary file 1 — Supplementary file1 (PDF 70 kb) [file 13205_2022_3434_MOESM1_ESM.pdf]

Table S1. Isolates used and results in evaluating this developed method

Taxon	Strain	Toxin product type	Detection result		
			tcdA	*tcdB*	*cdtB*
Escherichia coli	ATCC25922	N[a]	-	-	-
Enterococcus Faecom	ATCCBBA472	N	-	-	-
Enterococcus faecalis	ATCC51299	N	-	-	-
Clostridium perfringen	ATCC13124	N	-	-	-
Bacterooides fragilis	ATCC25285	N	-	-	-
Clostridium botulinum	Clinical isolates	N	-	-	-
C.difficile (Standard strains)	ATCCBBA1803	$A^{+}B^{+}cdtB^{+}$	+	+	+
	ATCC43594	$A^{+}B^{+}cdtB^{-}$	+	+	-
	ATCC43255	$A^{+}B^{+}cdtB^{-}$	+	+	-
	ATCC43593	$A^{-}B^{-}cdtB^{-}$	-	-	-
	ATCC43603	$A^{-}B^{-}cdtB^{-}$	-	-	-
	ATCC43598	$A^{-}B^{+}cdtB^{-}$	-	+	-
	HL017	$A^{-}B^{+}cdtB^{-}$	-	+	-
	ATCC9689	$A^{+}B^{+}cdtB^{-}$	+	+	-
	2	$A^{-}B^{+}cdtB^{-}$	-	+	-
	4	$A^{-}B^{+}cdtB^{-}$	-	+	-
	5	$A^{-}B^{+}cdtB^{-}$	-	+	-
	6	$A^{-}B^{+}cdtB^{-}$	-	+	-
	7	$A^{-}B^{+}cdtB^{-}$	-	+	-
	16	$A^{-}B^{+}cdtB^{-}$	-	+	-
	23	$A^{-}B^{+}cdtB^{-}$	-	+	-
	28	$A^{-}B^{+}cdtB^{-}$	-	+	-
	29	$A^{-}B^{+}cdtB^{-}$	-	+	-

	38	$A^{-}B^{+}cdtB^{-}$	-	+	-
	50	$A^{-}B^{+}cdtB^{-}$	-	+	-
	GZ2	$A^{-}B^{+}cdtB^{-}$	-	+	-
	GZ3	$A^{-}B^{+}cdtB^{-}$	-	+	-
	GZ6	$A^{-}B^{+}cdtB^{-}$	-	+	-
	GZ8	$A^{-}B^{+}cdtB^{-}$	-	+	-
	GZ11	$A^{-}B^{+}cdtB^{-}$	-	+	-
	GZ12	$A^{-}B^{+}cdtB^{-}$	-	+	-
	GZ13	$A^{-}B^{+}cdtB^{-}$	-	+	-
	GZ14	$A^{-}B^{+}cdtB^{-}$	-	+	-
	HN9	$A^{-}B^{+}cdtB^{-}$	-	+	-
	ZR8	$A^{-}B^{+}cdtB^{-}$	-	+	-
	ZR9	$A^{-}B^{+}cdtB^{-}$	-	+	-
	ZR18	$A^{-}B^{+}cdtB^{-}$	-	+	-
	ZR29	$A^{-}B^{+}cdtB^{-}$	-	+	-
	ZR58	$A^{-}B^{+}cdtB^{-}$	-	+	-
	ZR59	$A^{-}B^{+}cdtB^{-}$	-	+	-
	ZR65	$A^{-}B^{+}cdtB^{-}$	-	+	-
	ZR66	$A^{-}B^{+}cdtB^{-}$	-	+	-
	ZR68	$A^{-}B^{+}cdtB^{-}$	-	+	-
C.difficile	ZR72	$A^{-}B^{+}cdtB^{-}$	-	+	-
(clinical isolates)	ZR73	$A^{-}B^{+}cdtB^{-}$	-	+	-
	ZR82	$A^{-}B^{+}cdtB^{-}$	-	+	-
	BJ08	$A^{-}B^{+}cdtB^{-}$	-	+	-
	11034	$A^{+}B^{+}cdtB^{+}$	+	+	+
	12038	$A^{+}B^{+}cdtB^{+}$	+	+	+
	10122	$A^{-}B^{-}cdtB^{-}$	-	-	-
	10005	$A^{-}B^{-}cdtB^{-}$	-	-	-
	11032	$A^{-}B^{-}cdtB^{-}$	-	-	-
	25058	$A^{+}B^{+}cdtB^{+}$	+	+	+
	25053	$A^{+}B^{+}cdtB^{-}$	+	+	-
	10007	$A^{-}B^{-}cdtB^{-}$	-	-	-
	01047	$A^{+}B^{+}cdtB^{-}$	+	+	-

	20086	$A^-B^-cdtB^-$	-	-	-
	0201-018	$A^-B^+cdtB^-$	-	+	-
	0201-029	$A^+B^+cdtB^-$	+	+	-
	0201-041	$A^-B^+cdtB^-$	+	+	-
	0201-080	$A^+B^-cdtB^-$	+	-	-
	0203-004	$A^+B^+cdtB^-$	+	+	-
	0203-006	$A^-B^+cdtB^-$	-	+	-
	0205-001	$A^+B^-cdtB^-$	+	-	-
	0205-008	$A^-B^+cdtB^-$	-	+	-
	0206-003	$A^+B^+cdtB^-$	+	+	-
	0207-003	$A^+B^+cdtB^-$	+	+	-
	0207-006	$A^+B^+cdtB^-$	+	+	-
	0208-002	$A^-B^-cdtB^-$	-	-	-
	0208-003	$A^+B^-cdtB^-$	+	-	-
	0201-016	$A^+B^+cdtB^-$	+	+	-
	0201-021	$A^+B^-cdtB^-$	+	-	-
	0201-033	$A^+B^+cdtB^-$	+	+	-
	0201-045	$A^+B^+cdtB^-$	+	+	-
	0201-059	$A^+B^+cdtB^-$	+	+	-

a.N, not applicable.
