# Supplementary material for: A rapid multiplex real-time PCR detection of toxigenic Clostridioides difficile directly from fecal samples
Source: 3 Biotech. 2023 Jan 19;13(2):54. doi: 10.1007/s13205-022-03434-6 (PMC9849642; doi:10.1007/s13205-022-03434-6)
Supplement: Supplementary file 2 — Supplementary file2 (PDF 57 kb) [file 13205_2022_3434_MOESM2_ESM.pdf]

Table S2. The repeatability of this qPCR method within and between groups

Target gene	Concentration (copies/μL)	n	variation within groups			variation between groups		
			$\bar{x}$	SD	CV%	$\bar{x}$	SD	CV%
tcdA	5.53×10^2	3	31.315	0.139	0.44%	31.723	0.576	1.80%
	5.53×10^5	3	21.175	0.041	0.09%	21.578	0.647	2.99%
	5.53×10^7	3	16.093	0.012	0.07%	16.023	0.479	2.90%
tcdB	4.03×10^2	3	30.384	0.039	0.13%	33.369	0.476	1.42%
	4.03×10^5	3	22.815	0.058	0.25%	23.225	0.465	2.00%
	4.03×10^7	3	17.079	0.180	1.00%	17.026	0.038	0.22%
cdtB	4.28×10^2	3	31.897	0.176	0.55%	32.696	0.743	2.27%
	4.28×10^5	3	22.704	0.131	0.57%	22.685	0.049	0.21%
	4.28×10^7	3	15.144	0.140	0.92%	15.690	0.389	2.47%
