# Supplementary material for: A rapid multiplex real-time PCR detection of toxigenic Clostridioides difficile directly from fecal samples
Source: 3 Biotech. 2023 Jan 19;13(2):54. doi: 10.1007/s13205-022-03434-6 (PMC9849642; doi:10.1007/s13205-022-03434-6)
Supplement: Supplementary file 3 — Supplementary file3 (PDF 63 kb) [file 13205_2022_3434_MOESM3_ESM.pdf]

Table S3. Strains used to evaluate *tcdA* and comparison among three detected methods

Taxon	Strain	Detection result		
		tcdA		
		Lemee	Kato	our *tcdA*
Escherichia coli	ATCC25922	-	-	-
Enterococcus Faecom	ATCCBBA472	-	-	-
Enterococcus faecalis	ATCC51299	-	-	-
Clostridium perfringen	ATCC13124	-	-	-
Bacterooides fragilis	ATCC25285	-	-	-
Clostridium botulinum	Clinical isolates	-	-	-
C.difficile(Standard strains)	ATCCBBA1803	+	+	+
	ATCC43594	+	+	+
	ATCC43255	+	+	+
	ATCC43593	-	-	-
	ATCC43603	-	-	-
	ATCC43598	-	-	-
	HL017	-	-	-
	ATCC9689	+	+	+
C.difficile (RT017)	2	-	-	-
	4	-	-	-
	5	-	-	-
	6	-	-	-
	7	-	-	-
	16	-	-	-
	23	-	-	-
	28	-	-	-
	29	-	-	-
	38	-	-	-
	50	-	-	-
	GZ2	-	-	-
	GZ3	-	-	-
	GZ6	-	-	-
	GZ8	-	-	-
	GZ11	-	-	-
	GZ12	-	-	-
	GZ13	-	-	-
	GZ14	-	-	-
	HN9	-	-	-
	ZR8	-	-	-
	ZR9	-	-	-
	ZR18	-	-	-
	ZR29	-	-	-
	ZR58	-	-	-
	ZR59	-	-	-

	ZR65	-	-	-
	ZR66	-	-	-
	ZR68	-	-	-
	ZR72	-	-	-
	ZR73	-	-	-
	ZR82	-	-	-
	BJ08	-	-	-
	XA15	+	+	+
	Z132	+	+	+
	YN34	+	+	+
	YN13	-	-	-
	N20	-	+	+
	YN206	+	+	+
	18SD148	-	-	-
	18SD133	+	+	+
	18SD67	+	+	+
	18SD62	+	+	+
	18SD42	+	+	+
	18SD41	+	+	+
	XA4	-	-	-
	18SC199	+	+	+
C.difficile (Clinical isolates)	18SC207	+	+	+
	XA5	-	-	-
	18SC198	+	+	+
	XA8	+	+	+
	18SD140	+	+	+
	18SD145	+	+	+
	18SD110	-	-	-
	18SD178	+	+	+
	18SC139	+	+	+
	18SC152	-	-	-
	18SC135	+	+	+
	18SD195	+	+	+
	18SD18	+	+	+
	18SD36	-	-	-
	18SD25	+	+	+
	18SC197	+	+	+
